# Nonshaven Follicular Unit Extraction with Pretrimming Method and a Large-Session Hair Transplantation: Experience of a Brazilian Surgical Team

**DOI:** 10.1093/asjof/ojag144

**Published:** 2026-07-09

**Authors:** Maria Angélica Muricy, Dário Júnior de Freitas Rosa, Fabia Oppido Schalch

## Abstract

**Background:**

Androgenetic alopecia is a prevalent condition, with advanced cases often requiring surgical intervention. The nonshaven follicular unit extraction (nonshaven FUE) technique, combined with a large-session hair transplantation, has emerged as a strategy to optimize aesthetic outcomes and reduce social stigma.

**Objectives:**

The aim of this study was to describe the technique of nonshaven FUE with large-session hair transplantation using the pretrimming method.

**Methods:**

A retrospective analysis of medical records was conducted on patients who underwent hair transplantation using nonshaven FUE with individualized pretrimming of donor hairs. All procedures were performed by the same surgical team between May 2015 and October 2023.

**Results:**

The retrospective analysis was conducted on 236 patients (91.95% male; mean age 44.17 ± 10.12 years). A large-session hair transplantation (>2500 grafts) was successfully performed in all patients, with efficient donor harvesting and implantation in both men and women.

**Conclusions:**

Nonshaven FUE with pretrimming method and a large-session hair transplantation represent a safe and effective approach for advanced androgenetic alopecia.

**Level of Evidence: 4 (Therapeutic):**

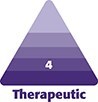

Androgenetic alopecia is one of the most common causes of medical consultation among men and women presenting with hair loss complaints.^1^ In advanced stages, or in cases unresponsive to conventional clinical treatment, there has been a significant increase in the demand for hair transplantation surgery in recent years.^1,2^

Currently, the most widely used technique is follicular unit extraction (FUE), considered a modern microsurgical approach in which follicular units (FUs) are harvested individually using specific surgical instruments known as punches from the donor area, and subsequently implanted one by one into the recipient area.^3,4^ However, one of the main limitations of this method—particularly for women—is the requirement for complete or partial shaving of the donor region.^4-6^

With the evolution of techniques, the nonshaven FUE approach has recently emerged, eliminating the need for full trichotomy and allowing patients to maintain their usual hair length. This strategy makes the postoperative recovery process less noticeable and more socially acceptable. In practice, scalp preparation occurs on the day before surgery, when a fraction of the selected hairs for extraction are randomly trimmed with scissors. On the following day, during the procedure, only the previously shortened follicles are removed, whereas the remaining hair length is preserved.^7^

Another relevant aspect is the increasing demand for surgical treatment among patients with advanced androgenetic alopecia. In this context, so-called “large-session hair transplantation” has become common, characterized by the extraction and implantation of more than 2500 FUs and more than 5000 hair shafts in a single procedure. In turn, the extraction of more than 4000 FUs characterizes megasessions. Extracting a large number of FUs in a single surgery thereby reducing the need for subsequent surgeries.^8,9^

Within this framework, the association between nonshaven FUE with pretrimming and a large-session hair transplantation represents an innovative and promising approach in hair restoration. The objective of this study was to describe the experience of a Brazilian surgical team in performing a large-session hair transplantation using the nonshaven FUE technique.

## METHODS

### Surgical Technique: Nonshaven FUE with Pretrimming Method

#### Surgical Planning

All patients underwent a preoperative consultation on the day before surgery. At this time, macroscopic photographs of the donor and alopecic areas were obtained. The design and placement of the frontal hairline were discussed and subsequently marked on the scalp, and the priority recipient regions were defined. Donor density was analyzed by visual inspection and dermatographic demarcation.

Patients received detailed postoperative care instructions and prescriptions for pain, edema, and inflammation control.

#### Donor Area Preparation

**Figure 1.**
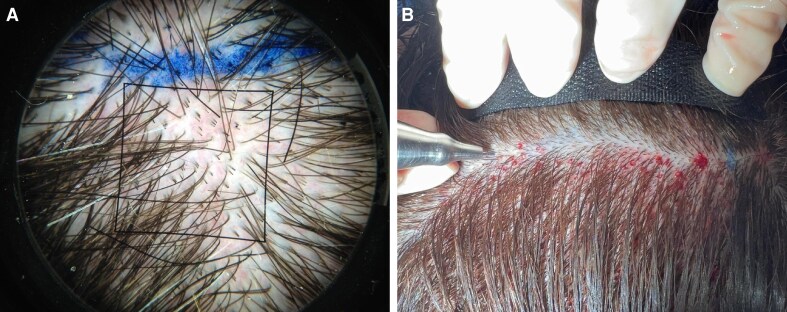
Methodological aspects. Woman, 60 years old, total of 2898 follicles. (A) Preoperative appearance of pretrimming area. (B) Extraction of pretrimmed follicular units.

Following the preoperative consultation and marking of donor and recipient areas, the selected hairs for extraction were trimmed among the long hair using fine-tipped scissors, leaving ∼2 mm in length (Figure 1A). The procedure was performed by 2 to 3 surgical assistants simultaneously, using magnifying loupes and a delicate comb. Typically, ∼20% more hairs than necessary were trimmed to facilitate subsequent extraction. Preparation took an average of 3 h for harvesting about 4000 FUs. During this process, the patient remained in the prone position with the head supported on the surgical table.

#### Patient Positioning

During surgery, each patient initially remained in the prone position for FUE, which lasted an average of 3 to 4 h, and was subsequently repositioned to the supine position for implantation.

#### Local Anesthesia with Sedation

After positioning on the surgical table, peripheral venous access was obtained, and intravenous sedation was initiated with midazolam 1 mg/mL, propofol 10 mg/mL, ketamine 50 mg/mL, fentanyl 0.0785 mg/mL and clonidine 150 µg/mL. A ring block of the donor area was then performed using 10 mL of ropivacaine 7.5 mg/mL. In the central region, a tumescent solution composed of 200 mL of saline, 20 mL of 2% lidocaine without vasoconstrictor, 1 mL of adrenaline 1 mg/mL, and 2 mL of triamcinolone hexacetonide 20 mg/mL was infiltrated.

In the recipient area, after repositioning, supratrochlear and supraorbital nerve blocks were performed with ropivacaine, in addition to infiltration of the same tumescent solution, except without adrenaline.

#### Surgical Procedure

FUE was initially performed using a manual punch and later replaced with a motorized device to reduce surgical time. Punches measuring 0.9 mm were used in the first cases, gradually reduced to the current use of flared punches ranging from 0.7 to 0.8 mm, depending on hair shaft thickness, in order to minimize the risk of hypochromic circular scars. Punches ≥0.85 mm were rarely used.

Up until September 2021, 6× magnification loupes were used; after this date, 8× loupes were adopted for both extraction and implantation stages.

The donor area was divided into quadrants (right and left temporal, right and left parietal, and occipital). Extraction was performed sequentially, from the right temporal to the occipital region. Scoring was performed from bottom to top using a modified Velcro device, as described by Carman and Schambach.^10^ Each quadrant was counted separately. During scoring, the surgeon performed the perforation, whereas the assistant extracted the FUs using 2 forceps, ensuring complete removal (Figure 1B).

Grafts were cleaned, examined under a Mantis Elite microscope (Vision Engineering, Surrey, United Kingdom) 6× magnification, sorted by hair count, and organized according to extracorporeal time, prioritizing the implantation of the earliest harvested units. All grafts were preserved at 4 °C in Ringer's lactate solution.

For implantation, preincisions were made using 21G needles, followed by the use of the Lion Implanter system (Hans Biomed, Seoul, South Korea) with a blunt needle. During this stage, the patient remained in the supine position.

### Methods and Data Analysis

A retrospective review was conducted of the medical records of 236 male and female patients who underwent nonshaven FUE with a large-session hair transplantation from May 2015 to October 2023 at Clínica Muricy, São Paulo.

The patients were divided into 3 groups. Initially, one subset comprised the male group (MG) and the other the female group (FG). Subsequently, the data were combined to form a group encompassing all evaluated patients (total group, TG).

The collected data included the date of surgery, sex, age at the time of transplantation, severity of baldness, surgical technique, total number of transplanted FUs, and number of hairs extracted.

The severity of hair loss was classified according to the Ludwig scale (Grades I-III) for female patients and the Norwood–Hamilton scale (Stages I-VII) for male patients.

For data analysis, the data were initially described using absolute and relative frequencies (qualitative variables) and by measures of central tendency and dispersion, such as mean, standard deviation (SD), minimum, median, and maximum (quantitative variables).

Graphs and statistical analyses were performed using Microsoft Excel for Windows (Microsoft Corporation, Redmond, WA) and SAS System for Windows, Release version 9.4 (SAS Institute, Cary, NC).

The study received approval from the institution's ethics committee under protocol CAAE 83876524.0.0000.5488.

## RESULTS

**Figure 2.**
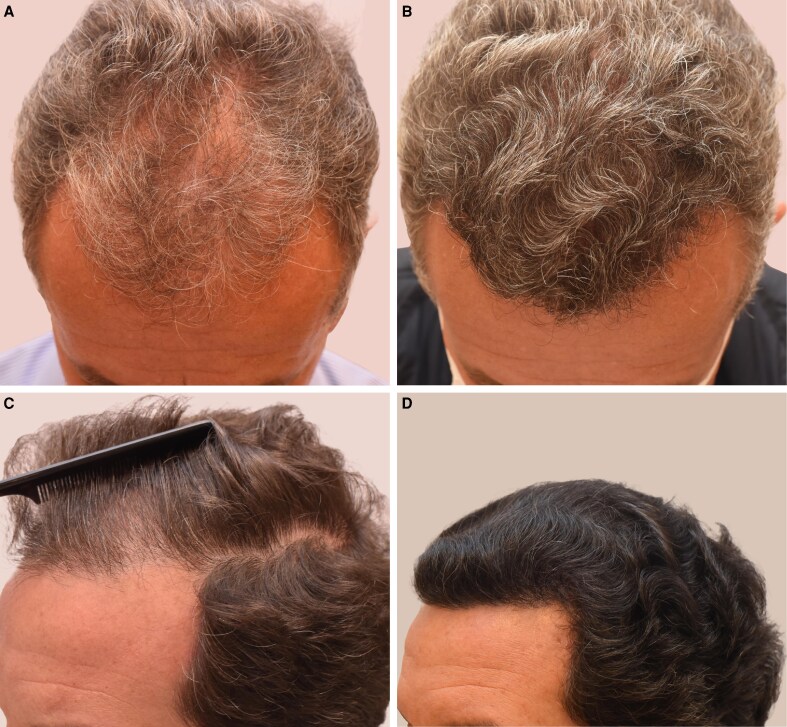
Clinical results of male patients undergoing hair transplantation in a superior and temporal view. Patient 1, male, 61 years old, 4007 transplanted follicles: (A) preoperative view; (B) after 12 months postoperative follow-up. Patient 2, male, 50 years old, 4017 transplanted follicles: (C) preoperative view; (D) after 13 months postoperative follow-up.

**Figure 3.**
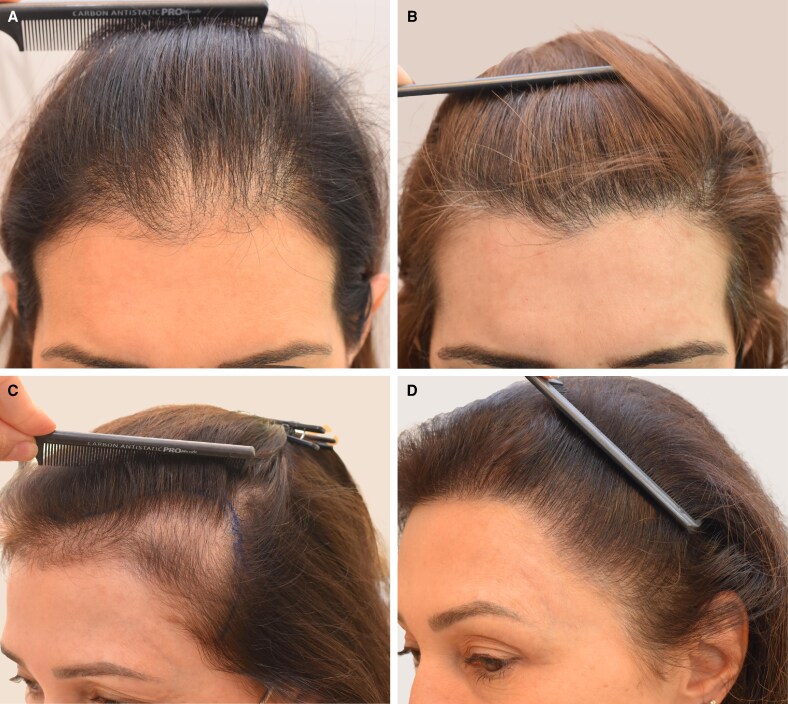
Clinical outcomes of female patients undergoing hair transplantation from a frontal and temporal perspective. Patient 3, woman, 57 years old, 4616 transplanted follicles, 2 sessions: (A) preoperative view; (B) after 14 months postoperative follow-up (second session) patient 4, woman, 66 years old, transplanted 2803 follicles: (C) preoperative view; (D) after 12 months postoperative follow-up.

A total of 236 patients underwent large-session hair transplantation using the nonshaven FUE technique with individualized pretrimming hairs. Patient ages ranged from 23 to 72 years, with a mean of 44.17 years (SD 10.12) and a median of 44.00 years (Q1 = 37; Q3 = 50). The sample consisted predominantly of males (91, 95%), with ages ranging from 23 to 71 years, whereas female patients ranged from 31 to 72 years (Figures 2, 3). All procedures were performed by the same surgical team between May 2015 and October 2023 (Tables 1, 2).

**Table 1.** Distribution of Nonshaven FUE Hair Transplantation Data by Group

	Female group	Male group
Age		
*n*	19	217
Mean (SD)	52.47 (12.79)	43.45 (9.55)
Median (Q1; Q3)	56 (40; 66)	43 (37; 49)
Minimum; maximum	31; 72	23; 71
Severity of baldness, *n* (%)		
1	0 (0)	1 (0.46)
2	14 (73.68)	31 (14.29)
3	5 (26.32)	46 (21.2)
4	0 (0)	44 (20.28)
5	0 (0)	45 (20.74)
6	0 (0)	46 (21.2)
7	0 (0)	4 (1.84)
Surgical time (h)		
*n*	14	197
Mean (SD)	8.39 (1.19)	8.24 (1,08)
Median (Q1; Q3)	8.25 (7.5; 9.33)	8.33 (7,5; 9)
Minimum; maximum	7; 11.08	5.5; 11.67
Missing = 25		
Total number of hair FUS		
*n*	19	217
Mean (SD)	3048.58 (417.08)	3663.16 (502.87)
Median (Q1; Q3)	2891 (2803; 3086)	3656 (3268; 4032)
Minimum; maximum	2560; 4005	2565; 5038
Total number of hairs		
*n*	19	217
Mean (SD)	6213.05 (937.23)	7,149,31 (1060.87)
Median (Q1; Q3)	5914 (5688; 6232)	7084 (6407; 7859)
Minimum; maximum	5070; 8680	5013; 10,218

FUs, follicular units; FUE, follicular unit extraction; SD, standard deviation.

**Table 2.** Clinical and Surgical Profile of All Patients Undergoing Nonshaven FUE

	Total
Age	
*n*	236
Mean (SD)	44.17 (10.12)
Median (Q1; Q3)	44 (37; 50)
Minimum; maximum	23; 72
Surgical time (h)	
*n*	211
Mean (SD)	8.25 (1.08)
Median (Q1; Q3)	8 (7.33; 8.67)
Minimum; maximum	4.17; 11.7
Missing = 25	
Total number of hair follicular units	
*n*	236
Mean (SD)	3613.68 (523.3)
Median (Q1; Q3)	3610 (3207; 4015)
Minimum; maximum	2560; 5038
Total number of hairs	
*n*	236
Mean (SD)	7073.94 (1080.23)
Median (Q1; Q3)	7049 (6185.5; 7797)
Minimum; maximum	5013; 10,218
Calculated mean density	
*n*	236
Mean (SD)	1.92 (0.18)
Median (Q1; Q3)	1.9 (1.78; 2.02)
Minimum; maximum	1.46; 2.39
Transection rate %	
*n*	234
Mean (SD)	4.24 (2.13)
Median (Q1; Q3)	4 (2.8; 5.6)
Minimum; maximum	0.02; 12
Missing = 2	

FUE, follicular unit extraction; SD, standard deviation.

The mean number of FUs transplanted in the FG was lower than in the MG, with averages of 3048.58 (SD 417.08) and 3663.16 (SD 502.87), respectively (Table 1). This difference was also reflected in the total number of hairs implanted, which was higher in the MG (mean 7149.31, SD 1060.87), representing a difference of more than 900 hairs compared with the FG (mean 6213.05, SD 937.23).

It is important to emphasize that 70 patients underwent transplantation with more than 4000 FUs, constituting a megasession of hair transplantation in 29.66% of patients.

The overall mean surgical time was 8.25 h (SD 1.08). In the early years of the series (2015 and 2016), surgeries were performed with longer surgical time, reflecting the team's learning curve. The mean operative time was 8.39 h (SD 1.19) among women and 8.24 h (SD 1.08) among men.

Analysis also revealed that 5 female patients did not have a diagnosis of female pattern hair loss but instead underwent the procedure with the specific goal of lowering the frontal hairline. In these cases, fewer than 2700 FUs were used, yet the aesthetic results were satisfactory. Additionally, the nonshaven FUE technique was applied to 1 female patient for the correction of a temporal scar, also achieving favorable clinical outcomes.

The mean calculated density was 1.92 (SD 0.18), which represents the average number of hairs transplanted per graft considering all patients, and the mean transection rate (TR) was 4.24% (SD 2.13).

## DISCUSSION

The requirement of complete scalp shaving remains one of the main barriers to performing FUE in candidates for hair transplantation.^11^ In response to this limitation, different approaches have been developed to minimize or eliminate visible shaved areas.^12,13^

Among FUE alternatives, partial shaving may be feasible when the hair is long enough to cover the donor area. However, this technique presents limitations in individuals with short hair or in professional and social contexts where partially shaved areas are not acceptable. Moreover, in cases requiring a large number of FUs, concentrated harvesting in a limited area may lead to perceptible local thinning.^14-16^

Another strategy is strip shaving, which, although more discreet, allows for the extraction of fewer FUs and also results in reduced density in the donor regions.^16^

Among the most recent techniques, nonshaven FUE stands out as a promising approach, particularly for patients with long hair.^13,17,18^ This modality requires longer surgical time and a steeper learning curve, potentially becoming physically and mentally demanding for the surgical team in very extensive cases, to FU-based procedure.^14-16^

Nevertheless, nonshaven FUE presents important advantages: it reduces the risk of visible scarring, preserves homogeneous donor density, and enables to megasessions with more than 4000 FUs in a single session.^18,19^ These characteristics, however, demand technical expertise and experience from the surgical team because of the procedure's complexity.^16^

**Figure 4.**
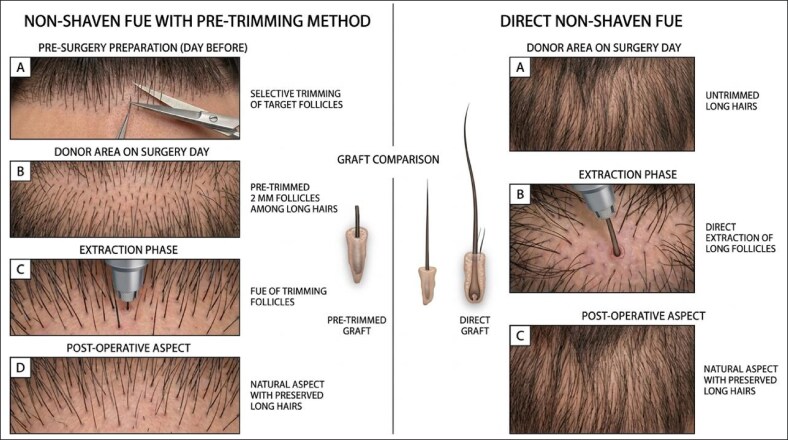
Comparative diagram of nonshaven follicular unit extraction (FUE) techniques: pretrimming vs conventional method or long hair FUE. During the preparation of this comparative diagram, the authors used the Gemini 3 Flash model (Google DeepMind, Mountain View, CA). After using this service, the authors reviewed and edited the content as needed and take full responsibility for the content of the publication.

There are 2 main variants of the technique: the direct method and the pretrimming method. In the direct method, trimming and extraction of the FU occur simultaneously using a rotary device, without previous preparation. In contrast, the pretrimming method involves trimming the hairs selected for extraction, usually the day before the procedure, with fine scissors. In this study, the pretrimming technique was employed, maintaining hair length between 1 and 2 mm, consistent with the literature (Figure 4).^18,19^

Compared with the direct nonshaven FUE technique with long hair extraction, a significant advantage of this pretrimming method is the greater savings on surgical extraction equipment, because there is no loss of cutting capacity of the punches that is commonly caused by long hair strands.

The traditional direct nonshaven FUE technique requires greater dexterity and technical skill from the hair transplant surgeon, because the extraction of long hairs can make punch alignment difficult and there may be a greater risk of hair breakage. The nonshaven FUE technique with pretrimming, on the other hand, presents itself as an option with a shorter effective surgical time, although a visit the day before surgery is necessary, and results in better quality extracted follicles because of less hair cutting, especially when considered by less experienced hands. Furthermore, it can also serve as a preparatory technique for those who will dedicate themselves to direct nonshaved FUE.

Limitation of the pretrimming method is the requirement for evaluation on the day before surgery for the selection and preparation of FUs, necessitating the patient's attendance on 2 consecutive days. This additional demand may represent greater logistical complexity compared with the direct nonshaven FUE approach, in which trimming and extraction are performed during the same operative session. This difference may particularly affect patients traveling from other cities or countries, as well as high-volume surgical centers. Nevertheless, individualized pretrimming may contribute to greater technical predictability and improved control during the extraction process.

Reported average operative time in the literature varies according to the technique employed. Some authors estimate the extraction of ∼1000 grafts per hour, depending on the method used.^20^ In patients undergoing large-session hair transplantation with complete shaving, the average surgical duration ranges from 6 to 12 h, depending on graft number and case complexity.^21^ Di et al reported an operative time averaging 10.6 h for the extraction of 3705 FUs using conventional FUE.^11^ In the present study, using the individualized pretrimming method, operative time averaged 8.25 h for the extraction of 3613.68 FUs, corresponding to 7073.94 hairs.

The calculated average graft density was 1.92 hairs per FU (SD 0.18). Park reported densities of 2.15 hairs per graft with the direct technique and 2.23 hairs per graft with the pretrimming method, although larger punch diameters were used.^18^ These values are influenced by several factors, including surgical team experience, donor area characteristics, hair shaft thickness, and operative planning.^14,18^

The TR observed in this study (4.24%; SD 2.13) was lower than that reported by Onda et al (5.4% using a motorized sharp punch), Harris (6.14%), and Park (8.8% with the pretrimming method).^18,20,22^

In the group, no patients required a second session for corrections or improvement in density. In contrast, Li et al, observed the need for additional surgery in 19% of cases treated with large-session FUE procedures, still with high satisfaction rates.^21^

Although the study includes a large and significant number of patients who underwent transplantation using the described technique, its principal limitations should be acknowledged. These include its retrospective design, with the inherent possibility of missing data, the absence of a control group and the failure to perform a statistical analysis, because this article is primarily intended to describe the surgical technique. Additionally, the study was conducted at a single center by the same hair transplant team, and there was no objective assessment of follicular survival, clinical outcomes, or patient satisfaction.

## CONCLUSIONS

This study demonstrates that large-session hair transplantation using the nonshaven FUE technique with individualized pretrimming is a feasible and reproducible surgical approach in appropriately selected patients. The method enabled the transplantation of a high number of FUs.

The procedure exhibited a low mean TR and an acceptable operative time, even in extensive cases, supporting its technical safety when performed by an experienced surgical team. Furthermore, the absence of secondary procedures suggests satisfactory clinical outcomes in the majority of patients.

Although additional prospective and comparative studies are warranted, the present findings indicate that this innovative approach represents a safe and viable alternative for large-session hair transplantation, thereby expanding the surgical armamentarium available to hair restoration surgeons.
